# Impact of Common Dizziness Associated Symptoms on Dizziness Handicap in Older Adults

**DOI:** 10.3389/fneur.2021.801499

**Published:** 2021-12-17

**Authors:** Tino Prell, Alexander Wassermann, Hannah M. Zipprich, Sigrid Finn, Hubertus Axer

**Affiliations:** ^1^Department of Geriatrics, Halle University Hospital, Halle, Germany; ^2^Center for Healthy Ageing, Jena University Hospital, Jena, Germany; ^3^Department of Neurology, Jena University Hospital, Friedrich Schiller University, Jena, Germany; ^4^Center for Vertigo and Dizziness, Jena University Hospital, Jena, Germany

**Keywords:** dizziness, aged, vertigo, headache, visual problems, hearing loss

## Abstract

**Background:** A cross-sectional observational study was designed to determine the impact of dizziness associated symptoms on the dizziness handicap inventory (DHI) in older adults (≥60 years).

**Methods:** In total, 785 individuals referred to a multidisciplinary dizziness unit were assessed. Participants completed self-report questionnaires with general questions about symptoms of dizziness as well as the DHI. The DHI subscores (physical, functional, emotional) were calculated. Medical diagnoses were collected from the medical records of the patients. One-way MANOVA and networking analysis were used to analyze the impact of dizziness associated symptoms on dizziness handicap.

**Results:** Most patients reported swaying dizziness (60.6%) and feeling of unsteadiness (59.8%) with substantial overlap between the types of dizziness. Most frequent dizziness associated symptoms were ear noise/tinnitus, visual problems, and nausea/vomiting. Network analysis revealed that visual disturbances, headache, and hearing impairment were associated with higher DHI and explained 12% of the DHI variance in the linear regression. In the one-way MANOVA visual problems and headache had an effect on all three DHI subscores, while hearing impairment was associated with the functional and emotional subscores of DHI.

**Conclusion:** Distinct dizziness associated symptoms have substantial impact on dizziness handicap in older adults. A multifactorial assessment including these symptoms may assist in tailoring therapies to alleviate dizziness handicap in this group.

## Introduction

Vertigo and dizziness are common complaints in the general population (1). According to the International Bárány Society for Neuro-Otology, vertigo and dizziness can be distinguished in that way that “vertigo is the sensation of self-motion when no self-motion is occurring; dizziness is the sensation of disturbed or impaired spatial orientation without a false or distorted sense of motion; and imbalance or unsteadiness is the feeling of being unstable while seated, standing, or walking without a particular directional preference” (2). Dizziness may have various causes and can occur in peripheral, central, and “higher” vestibular disorders. It is more common in women and in older adults and has a profound effect on daily functioning and health-related quality of life (3). However, symptoms of dizziness and vertigo often are described rather imprecisely and vague and symptoms often overlap (4).

The incidence of dizziness increases with older age (5) and leads to physical inactivity (6) and disability (7), a higher risk of falls (8), and social isolation and depression (9). Thus, dizziness results in a significant increase of functional impairment in daily life activities. The Dizziness Handicap Inventory (DHI) was developed to quantify the self-reported impact of dizziness on daily life (10). Mean DHI score was 5.6 ± 11.2, consistent with the absence of self-reported dizziness handicap in healthy people 70 years and older (11). In patients with vestibular disorders aged 65 years or higher, the DHI score has been shown to have a significant association with the Timed Up and Go test and usual gait speed using the timed 10-meter walk test (12). Older patients with dizziness score higher in DHI than younger patients (13–15) and also multimorbidity is correlated to higher DHI scores (16).

Dizziness and vertigo are often associated with additional symptoms, such as headache, visual problems, or nausea. Little is known about these dizziness associated symptoms (DAS) and their association to dizziness handicap. Therefore, the aim of this study was to investigate the relationship between DAS and DHI in older adults referred to a multidisciplinary dizziness unit. Here, patients with chronic dizziness or vertigo were assessed with symptoms lasting over more than 3 months. Dizziness and vertigo is not the same, but patients' description of vertigo and dizziness often is not suited to assign them to vestibular and non-vestibular origin (15).

## Methods

### Patients

During the study period, a total of 3,216 patients visited the specialized Center for Vertigo and Dizziness at Jena University Hospital between January 2013 and November 2017. The Center for Vertigo and Dizziness is a multidisciplinary tertiary care outpatient clinic which provides multidisciplinary diagnosis of patients with chronic vertigo and dizziness and offers multimodal and interdisciplinary day care treatment programs (17). It is an interdisciplinary project of the departments of neurology, ENT, and physiotherapy at the University Hospital. As the patients seen here are composed of all age groups, there is no specific geriatric specialist regularly available. Diagnoses are generally based on the International Classification of Vestibular Disorders of the Bárány Society.

The study was approved by the local ethics committee (Ethics Committee of the Friedrich-Schiller-University Jena, Number 5426-02/18) and written informed consent for study participation was obtained from all patients.

Dizziness and vertigo were defined to be chronic if symptoms persisted at least for 3 months or attacks recurred often in the last 3 months (≥5 days with symptoms/month). Criteria for patient recruitment were: patients suffered from chronic dizziness and/or vertigo, age ≥ 60 years, consent for study participation and completed questionnaire. The majority of these patients had preserved mobility and the patients were able to walk independently.

Patients suffering from chronic dizziness and vertigo were asked to complete a questionnaire at their first appointment. The patients had to indicate whether they agreed to allow their anonymized data to be analyzed for the study. The questionnaire included age, gender, general questions about symptoms of dizziness, as well as the DHI. In addition, duration of dizziness in years, the frequency of dizziness, and category of dizziness (physical and functional), and DAS (i.e., ear pressure, hearing impairment, headache, nausea/vomiting, visual problems, and ear noise/tinnitus) were assessed.

The DHI was designed in 1990 to quantify the self-perceived handicap due to dizziness (10). It contains 25 questions; a *yes* response yields a score of 4 points, *sometimes* 2 points, and *no* 0 points. The scale consists of a 7-item physical subscale (maximum score 28), a 9-item emotional subscale (maximum score 36) and a 9-item functional subscale (maximum score 36).

Totally 1,752 patients filled out the questionnaire and agreed to take part in the study. Some aspects of the analysis of these questionnaires have already been published elsewhere (15). For this study, we selected 785 questionnaires from those patients with chronic dizziness/vertigo aged 60 years and older.

### Analyses

All data were analyzed with the Statistical Package for the Social Sciences software (version 25.0; IBM Corporation, USA) Jamovi (1.8.2.0) or JASP 0.14.1.0. The values were presented as mean and standard deviation (SD) or numbers and percentages. Normal distribution was determined using the Shapiro–Wilk-test. First, we described the cohort using descriptive statistics. Nested linear regression was used to determine the association between DHI and clinical variables and DAS (after exclusion of multicollinearity and autocorrelation). Multivariate analysis of variance (MANOVA) was used to study the effects of DAS on DHI subscores. Correlations between dependent variables were low (*r* < 0.90), indicating that multicollinearity was not a confounding factor in the analysis. One multivariate outlier was found using the Mahalanobis distance (*p* > 0.001) and not removed from analysis. The presence or absence of this outlier did not affect the results. Homogeneity of the error variances was assessed using Levene's-test. Homogeneity of covariances was noted as assessed using Box's-test. Multivariate analysis of covariance (MANCOVA) was used to adjust these findings for age, gender and other clinical variables.

A network model was calculated to explore the association between DHI and DAS. A network model conceptualizes factors as a network of mutually interacting characteristics. The network was estimated with JASP based on the mgm and qgraph R-packages according to the methods described (18–20). The network analysis was conducted on the following variables: DHI total score, visual problems, ear noise/tinnitus, ear pressure, headache, hearing impairment, and nausea/vomiting. Within the networks, variables are represented as nodes (circles; observed variables), connected by edges (lines; relations between variables). Edges are the regularized partial correlations between the nodes. Nodes are reciprocally connected. Here, we used lasso regularization and graphical lasso to control for spurious connections that may arise due to multiple testing (21, 22). In the resulting sparse GGM many edge weights are set to zero and thus removed from the network. Therefore, the network is interpretable and guarded against overfitting. We controlled the sparsity of the network by minimizing the Extended Bayesian Information Criterion (23). The measure of closeness centrality was used to analyze position and function of items within the network (24). In the network model the stronger the edge (path = edges between nodes), the stronger the path between relevant nodes, and the easier it is to travel from one node to another (19). The more central the node is, the easier it is to travel to all other nodes. Therefore, nodes with high closeness centrality have a high ability to predict other nodes and are important in the network.

For all analyses a *p*-value <0.05 was considered statistically significant.

## Results

Patients were 71.7 ± 7.2 years old (range: 60–90 years), 60.4% were female. Patients suffered from dizziness/vertigo since 5.0 ± 7.2 years (range 0.04–79 years). The patient with the longest time course of symptoms was an 85 year old woman with the diagnosis of vestibular migraine who reported that she had her first episode of spinning vertigo with 6 years of age. The mean DHI was 49.5 ± 20.5, with highest values in the functional DHI (*M* = 19.4 ± 9.1), followed by emotional DHI (*M* = 15.8 ± 8.5) and physical DHI (*M* = 14.2 ± 6.9). Characteristics of dizziness and underlying main diagnoses are given in Table 1.

**Table 1:** Characteristic of the cohort, *n* = 785.

	***n* (%)**
**Frequency of dizziness/vertigo**	
• Less than once a month	51 (6.7%)
• Several times a month	104 (14%)
• Several times a week	138 (18%)
• Daily	467 (61%)
• Unknown	25
**Duration of dizziness/vertigo**	
• Days	244 (34%)
• Hours	213 (30%)
• Minutes	155 (22%)
• Seconds	100 (14%)
• Unknown	73
**Type of symptoms**	
• Spinning vertigo (yes/no/unknown)	336 (44%)/436 (56%)/13
• Swaying vertigo (yes/no/unknown)	468 (61%)/304 (39%)/13
• Tilt vertigo (yes/no/unknown)	134 (17%)/638 (83%)/13
• Feeling of unsteadiness (yes/no/unknown)	462 (60%)/310 (40%)/13
**Diagnoses**	
• Persistent postural–perceptual dizziness (PPPD)	179 (23.4%)
• Benign paroxysmal positional vertigo	94 (12%)
• Unilateral peripheral vestibulopathy	62 (8.0%)
• Bilateral peripheral vestibulopathy	31 (4.0%)
• Meniere's disease	50 (6.4%)
• Vestibular schwannoma	9 (1.2%)
• Vestibular paroxysm	11 (1.4%)
• Central vestibular vertigo	126 (16%)
• Vestibular migraine	14 (1.8%)
• Multisensory deficit	191 (25%)
• Syncope	10 (1.3%)
• Unknown	8

**Figure 1:**
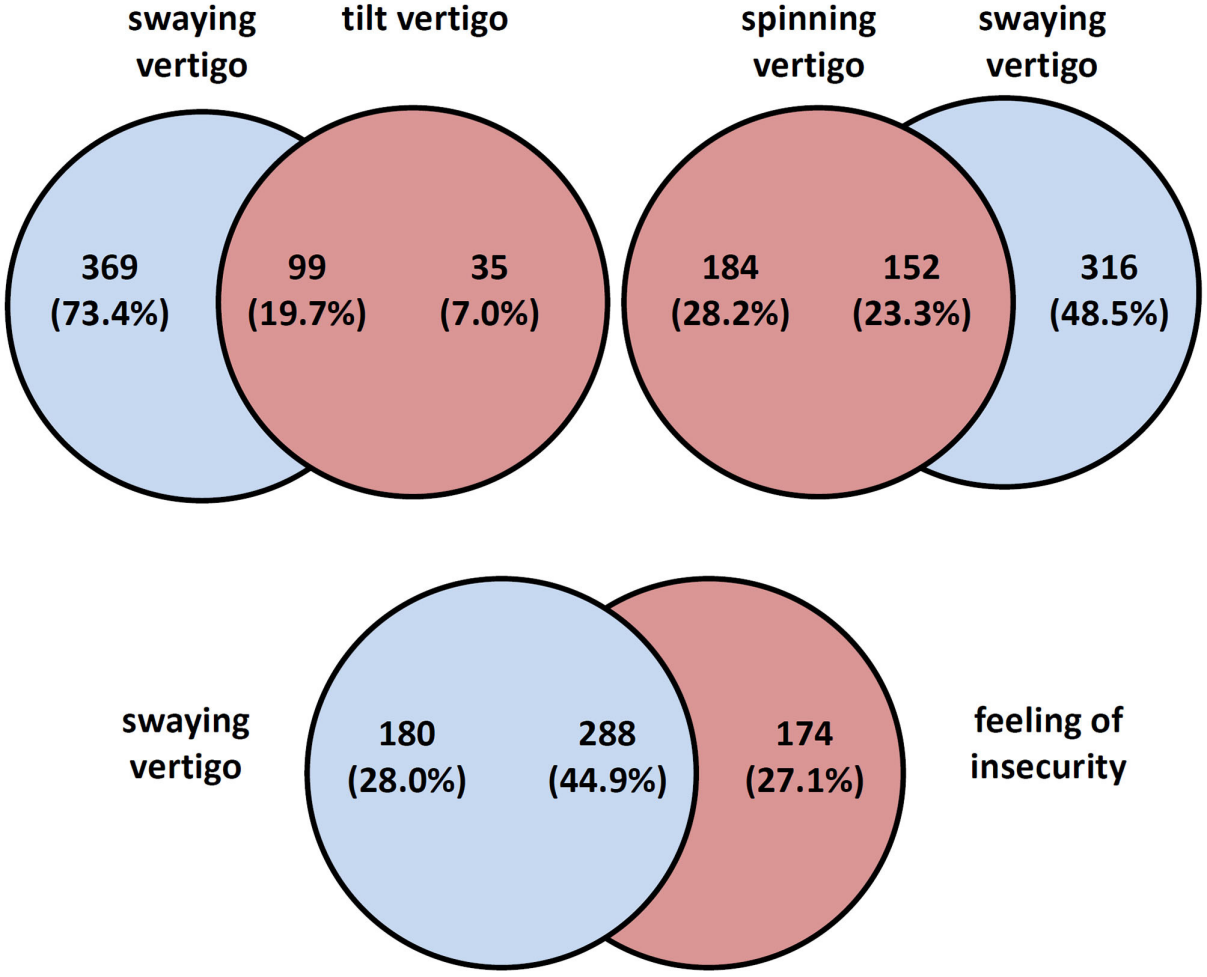
Venn diagrams: overlap between types of vertigo and dizziness.

**Figure 2:**
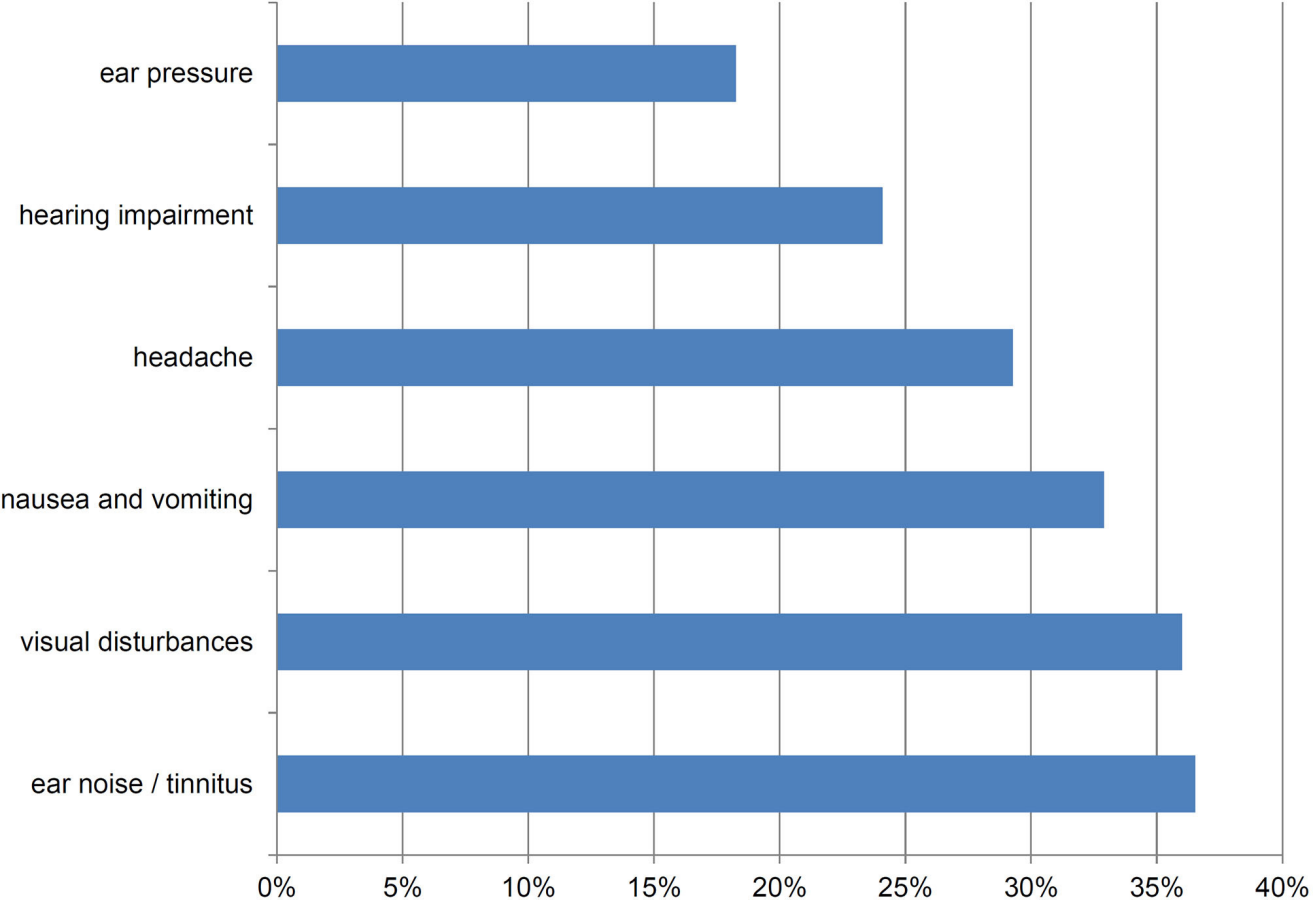
Frequency of dizziness-related symptoms (*n* = 772).

**Figure 3:**
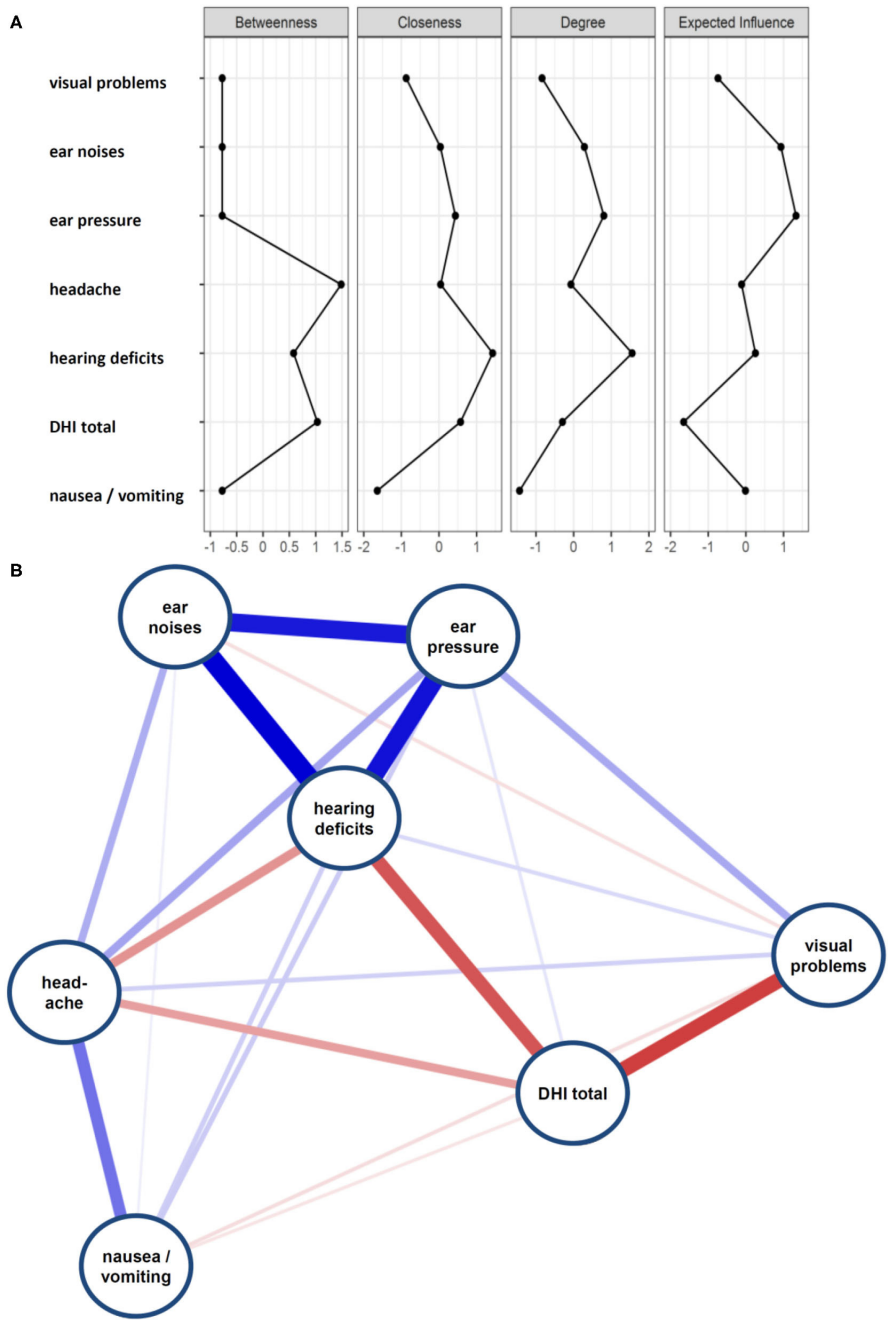
**(A)** Centrality plot of dizziness associated symptoms and DHI total score. **(B)** Network diagram on DHI and DAS.

Most patients reported swaying vertigo (*n* = 468, 60.6%) and feeling of unsteadiness (*n* = 462, 59.8%) followed by spinning vertigo (*n* = 336, 43.5%) and tilt vertigo (*n* = 134, 17.4%). The majority (59.6%) reported more than one type of dizziness and a substantial overlap between the types of dizziness was found (Figure 1). Among the studied DAS, most patients complained about ear noise/tinnitus and visual disturbances (Figure 2). Network analysis revealed that visual problems, headache, and hearing impairment were associated with higher DHI (Figure 3). These three symptoms together explained 12% of the DHI variance in the linear regression (Table 2). After entering age, gender, duration of dizziness in years, frequency of dizziness, and category of dizziness the explained variance increased to 25% (Table 2).

**Table 2:** Predictors of DHI: nested linear regression (Durbin Watson = 1.95).

**Predictor**	**Estimate**	**SE**	***t***	***P***	**Stand. estimate**	**Lower 95%CI**	**Upper 95%CI**
**Model 1 (Adjusted** ***R*^2^** **=** **0.1179), dependent variable** **=** **DHI total score**							
Intercept	68.8291	2.459	27.9858	<0.001			
Visual problems: No—Yes	−8.7639	1.610	−5.4448	<0.001	−0.42544	−0.5789	−0.27201
Hearing deficits: No—Yes	−7.3009	1.891	−3.8615	<0.001	−0.35442	−0.5346	−0.17419
Ear noises: No—Yes	−2.0086	1.699	−1.1819	0.238	−0.09750	−0.2595	0.06450
Ear pressure: No—Yes	−2.0907	2.093	−0.9987	0.318	−0.10149	−0.3010	0.09806
Headache: No—Yes	−8.0233	1.682	−4.7704	<0.001	−0.38948	−0.5498	−0.22916
Nausea/vomiting: No—Yes	0.2220	1.642	0.1352	0.892	0.01078	−0.1457	0.16725
**Model 2 (Adjusted** ***R*^2^** **=** **0.1618), dependent variable** **=** **DHI total score**							
Intercept	55.8954	7.9125	7.0641	<0.001			
Visual problems: No—Yes	−8.6565	1.5696	−5.5152	<0.001	−0.42022	−0.569839	−0.27060
Hearing deficits: No—Yes	−6.5613	1.8657	−3.5168	<0.001	−0.31851	−0.496358	−0.14067
Ear noises: No—Yes	−2.5041	1.6730	−1.4968	0.135	−0.12156	−0.281033	0.03792
Ear pressure: No—Yes	−1.2475	2.0562	−0.6067	0.544	−0.06056	−0.256567	0.13545
Headache: No—Yes	−7.0761	1.6488	−4.2917	<0.001	−0.34350	−0.500674	−0.18633
Nausea/vomiting: No—Yes	−0.4496	1.6478	−0.2728	0.785	−0.02182	−0.178901	0.13525
Sex: Male—Female	−8.3894	1.5335	−5.4708	<0.001	−0.40726	−0.553437	−0.26108
Age	0.2119	0.1097	1.9315	0.054	0.07319	−0.001220	0.14761
**Model 3 (Adjusted *R*^2^ = 0.2447), dependent variable** **=** **DHI total score**							
Intercept	54.94560	8.31431	6.6086	<0.001			
Visual problems: No—Yes	−7.38174	1.50574	−4.9024	<0.001	−0.35834	−0.501878	−0.21480
Hearing deficits: No—Yes	−5.24149	1.79327	−2.9229	0.004	−0.25444	−0.425390	−0.08350
Ear noises: No—Yes	−2.66541	1.60062	−1.6652	0.096	−0.12939	−0.281972	0.02319
Ear pressure: No—Yes	−0.70104	1.96532	−0.3567	0.721	−0.03403	−0.221379	0.15332
Headache: No—Yes	−6.75233	1.57517	−4.2867	<0.001	−0.32779	−0.477942	−0.17763
Nausea/vomiting: No—Yes	−4.59587	1.66851	−2.7545	0.006	−0.22310	−0.382156	−0.06405
Sex: Male—Female	−8.02903	1.46433	−5.4831	<0.001	−0.38976	−0.529352	−0.25017
Age	0.04018	0.10890	0.3690	0.712	0.01388	−0.059989	0.08775
**Vertigo category:**							
Psychological diagnoses—Somatic diagnoses	−0.60712	2.25546	−0.2692	0.788	−0.02947	−0.244476	0.18553
Non-specific diagnoses—Somatic diagnoses	−5.89410	2.21806	−2.6573	0.008	−0.28612	−0.497564	−0.07468
Duration of dizziness in years	0.09285	0.09939	0.9342	0.351	0.03233	−0.035625	0.10028
**Frequency of dizziness:**							
Several times a month—Less than once a month	6.53857	3.37100	1.9397	0.053	0.31741	−0.003935	0.63875
Several times a week—Less than once a month	8.29794	3.30791	2.5085	0.012	0.40282	0.087486	0.71815
Daily—Less than once a month	18.44261	3.04589	6.0549	<0.001	0.89528	0.604929	1.18564

We then focused on the impact of DAS on different DHI subscales. Here, a one-way MANOVA showed a statistically significant difference between headache and hearing impairment on the combined dependent variables (Supplementary Table 1). There were no significant interactions among these three symptoms. *Post-hoc* univariate ANOVAs were conducted for every dependent variable. Results show statistically significant difference between headache and hearing impairment on all three DHI subscales (Supplementary Table 1). After entering age, gender, duration of dizziness, and category of dizziness into the model, hearing impairment, headache, and additionally visual problems showed a statistically significant difference on the combined dependent variables (Supplementary Table 2). While visual problems and headache had an effect on all three DHI subscores, hearing impairment was associated with DHI functional and DHI emotional (Supplementary Table 2).

## Discussion

Our study population of patients with chronic dizziness aged 60 years and older showed a total DHI of 49.5 ± 20.5, indicating a moderate to severe dizziness related handicap. Another study of patients with vestibular disorders aged ≥ 65 years scored 47.3 ± 21.3 in the total DHI (12), which is in good concordance with our data. It mainly stresses the fact that chronic dizziness leads to significant impairment in the older population. In addition, we have previously demonstrated (14) that younger patients with chronic dizziness show lower DHI scores (41.5 ± 17.7 in an age group <41 years and 46.7 ± 20.3 in an age group between 41 and 65 years). Patients' descriptions of symptoms such as spinning vertigo, swaying vertigo, tilt vertigo, and a feeling of unsteadiness were not suited to make a clear diagnosis. Moreover, there was a large overlap between different types of self-reported symptoms (Figure 1), which also has been described in other studies (4).

The most common diagnosis was multisensory deficit in 25% of cases. The diagnosis multisensory deficit was given when deficits in more than one sensory system (vestibular, somatosensory, or visual, of central, or peripheral origin) were detectable. The relatively high amount of multisensory deficits in our patient population emphasizes the increase of degenerative etiologies in older adults. In our cohort, persistent postural–perceptual dizziness (PPPD) was diagnosed in 23.4%. In 34 from 179 PPPD cases (19%) triggering events could be identified. In comparison to younger age groups the portion of PPPD diagnoses is lower in the older population (17). Benign paroxysmal positional vertigo (BPPV) may be the most common vestibular diagnosis in the general population (25). It has been shown that BPPV is a common cause of dizziness among older adults (26, 27). In our study 12% of the patients had BPPV. This relatively small amount of BPPV diagnoses in our sample may be due to the fact that general practitioners nowadays know well to diagnose and treat BPPV. Thus, they are able to manage these patients by themselves. Patients are referred to our tertiary care center if symptoms become chronic. Especially in respect to increased risk of falls the detection of BPPV and its treatment is essential (28). Therefore, clinical testing for BPPV is routinely part of every clinical examination in our center.

Patients with specific geriatric diagnoses for dizziness such as orthostatic hypotension, medication side effects, cardiovascular problems, and others were not referred to the center with the exception of syncopes (in 1.3%). This is mainly reasonable as the outpatient clinic is not a specific geriatric institution and takes care of patients of all age groups. It has to be kept in mind that the patients analyzed here were outpatients with relatively good health status. They had preserved mobility and the majority of patients were able to walk independently. In addition, the distribution of diagnoses changes with age (15). Thus, the results of our study cannot be generalized to the general population.

The major proposition of our study is that dizziness associated symptoms influence the handicap due to dizziness (measured by DHI) to various degrees in the older population. In older adults, mainly hearing impairment, visual problems, as well as headache were found to be associated with DHI. Thus, these dizziness associated symptoms deserve some closer examination.

Hearing loss can be described as the quietest sound a person could hear on the better ear, and a grading from normal, over mild, moderate, severe to complete can be made according to audiometric thresholds (29). Hearing loss represents the most common sensory disturbance. About 1.57 billion people had hearing loss world-wide in 2019, which represents 20.3% of the global population (29). There is a clear association between hearing loss and age (29, 30). Hearing loss was most severe in people older than 70 years, and after the age of 50 years its prevalence increases continuously (29).

Tinnitus is another common comorbidity of the hearing system. Tinnitus is the perception of sound in the absence of external noise. About 80% of patients with tinnitus are older than 40 years with highest incidence rate in people between 60 and 69 years of age (31). The prevalence of tinnitus was 21.4% in adults older than 50 years (32). About 10% of the people with tinnitus had restrictions in daily life and hearing impairment was found twice likely in people with tinnitus (32). In people older than 40 years, tinnitus has been associated with age, quality of life, depressive mood, hearing loss, and dizziness (33). However, other studies did not find an association between tinnitus and age (34).

It has been shown that the different types of frailty may have divergent associations with hearing loss and tinnitus (35). Tinnitus has further been shown to significantly increase the risk of falling, in addition to age and generalized osteoarthritis (36). Furthermore, poor hearing acuity also induces a higher risk of falls (37). There is increasing evidence that hearing loss as well as tinnitus affect mental health and may be associated with cognitive impairment, stress, anxiety and depression (38).

The impairment of vision is a further significant complaint of older people. Its prevalence in people aged 60 years and older was 13% in Malaysia with an increasing trend in older age (39). In a population ≥50 years, another study found 14.6% with impaired vision only, 11.6% with impaired hearing only and 7.6% with both impaired vision and hearing (40). Visual impairment is associated with falls (41). It has been shown that the coincidence of vision and hearing impairment further increases the risk of falls (40). In addition, it leads to restricted daily activity (42) and is associated with increased risk for mild cognitive impairment (43) and faster cognitive decline (44). Visual impairment worsens frailty in the early stages of its development (45).

Headache is a common complaint in the general population. Headache prevalence decreases in patients older than 65 years and tension-type headache is the most common diagnosis in this population (46). In a population of US-Americans ≥ 50 years, 17.8% met headache criteria (47). Chronic headache in the elderly affects health-related quality of life (48) and is associated with anxiety and depressive symptoms (47).

In conclusion, hearing impairment, tinnitus, visual disturbances, and headache are common complaints in advanced age. It has been shown that these complaints affect a wide range of physical and mental health. The Berlin Aging Study has already demonstrated a strong relationship between sensory function and a broad range of daily life functions (49). Here, we were able to demonstrate that dizziness-associated complaints have an important impact on dizziness associated handicap in older people with chronic dizziness/vertigo.

Therefore, DAS must carefully be considered when studying dizziness handicap in older adults, even though older patients reported nausea, headache, tinnitus, and visual impairment less frequently than younger patients (14). The question of whether therapeutic attention to these dizziness associated symptoms may have an impact on improving dizziness related handicap in the elderly population will be an interesting focus of future research. Reasonable measures could be optimization of hearing aids, optimization of glasses, improvement of lightning, and treatment of headache.

Our study is not free of limitations. As mentioned above the findings cannot be generalized to the general population, as we focused on people with chronic dizziness/vertigo, which need specialized multidisciplinary treatment. By nature, not all possible cofactors could be assessed and included in the analyses. In particular, we cannot provide detailed data about comorbidities and validated questionnaires about the general health status.

## Data Availability Statement

The raw data supporting the conclusions of this article will be made available by the authors, without undue reservation.

## Ethics Statement

The studies involving human participants were reviewed and approved by Ethics Committee of the Friedrich-Schiller-University Jena, Number 5426-02/18. The patients/participants provided their written informed consent to participate in this study.

## Author Contributions

TP and HA: design of the study and writing of the paper. AW and SF: collection of data. TP, HA, and HZ: analysis. All authors critical revision of the article, contributed to the article, and approved the submitted version.

## Funding

TP was supported by a BMBF (Bundesministerium für Bildung und Forschung) Grant (01GY1804).

## Conflict of Interest

The authors declare that the research was conducted in the absence of any commercial or financial relationships that could be construed as a potential conflict of interest.

## Publisher's Note

All claims expressed in this article are solely those of the authors and do not necessarily represent those of their affiliated organizations, or those of the publisher, the editors and the reviewers. Any product that may be evaluated in this article, or claim that may be made by its manufacturer, is not guaranteed or endorsed by the publisher.
