# Supplementary material for: Impact of Common Dizziness Associated Symptoms on Dizziness Handicap in Older Adults
Source: Front Neurol. 2021 Dec 17;12:801499. doi: 10.3389/fneur.2021.801499 (PMC8718649; doi:10.3389/fneur.2021.801499)
Supplement: Supplementary file 1 [file Table_1.DOCX]

**Supplementary Table 1.** MANOVA: Association between DHI subscores and dizziness-related symptoms.

| **Multivariate tests** | | | | | | |
| --- | --- | --- | --- | --- | --- | --- |
| **Effect** | **Wilks-Lambda** | **F** | **Hypothesis df** | **Error df** | **p** | **partial η²** |
| Constant | 0.274 | 606.779 | 3 | 686 | .000 | .726 |
| Nausea / vomitus | 0.998 | 0.483 | 3 | 686 | .694 | .002 |
| Headache | 0.968 | 7.466 | 3 | 686 | .000 | .032 |
| Ear pressure | 0.998 | 0.467 | 3 | 686 | .706 | .002 |
| Ear noises | 0.994 | 1.325 | 3 | 686 | .265 | .006 |
| Hearing deficits | 0.988 | 2.813 | 3 | 686 | .039 | .012 |
| Visual problems | 0.990 | 2.303 | 3 | 686 | .076 | .010 |
| **Post-hoc univariate ANOVAs for every dependent variable (only significant effects reported)** | | | | | | |
|  | **Dependent variable** | | | **F** | **p** | **partial η²** |
| Corrected model | DHI-Physical-Total | | | 1.506 | .009 | .118 |
|  | DHI-Functional-Total | | | 2.238 | .000 | .166 |
|  | DHI-Emotional-Total | | | 1.995 | .000 | .150 |
| Headache | DHI-Physical-Total | | | 16.728 | .000 | .024 |
|  | DHI-Functional-Total | | | 17.284 | .000 | .025 |
|  | DHI-Emotional-Total | | | 7.001 | .008 | .010 |
| Hearing deficits | DHI-Physical-Total | | | 4.585 | .033 | .007 |
|  | DHI-Functional-Total | | | 7.341 | .007 | .011 |
|  | DHI-Emotional-Total | | | 4.687 | .031 | .007 |
| Visual problems | DHI-Physical-Total | | | 0.725 | .395 | .001 |
|  | DHI-Functional-Total | | | 6.573 | .011 | .009 |
|  | DHI-Emotional-Total | | | 2.087 | .149 | .003 |
