# Supplementary material for: Impact of Common Dizziness Associated Symptoms on Dizziness Handicap in Older Adults
Source: Front Neurol. 2021 Dec 17;12:801499. doi: 10.3389/fneur.2021.801499 (PMC8718649; doi:10.3389/fneur.2021.801499)
Supplement: Supplementary file 2 [file Table_2.DOCX]

**Supplementary Table 2.** MANOVA: Association between DHI subscores and dizziness related symptoms with additional covariates.

| **Multivariate tests** | | | | | | | |
| --- | --- | --- | --- | --- | --- | --- | --- |
| **Effect** | **Wilks-Lambda** | | **F** | **Hypothesis df** | **Error df** | **p** | **partial η²** |
| Constant | .942 | | 12.957 | 3.000 | 634.000 | .000 | .058 |
| Age | .994 | | 1.349 | 3.000 | 634.000 | .258 | .006 |
| Duration of dizziness (years) | .995 | | 1.128 | 3.000 | 634.000 | .337 | .005 |
| Nausea / vomitus | .988 | | 2.536 | 3.000 | 634.000 | .056 | .012 |
| Headache | .973 | | 5.877 | 3.000 | 634.000 | .001 | .027 |
| Ear pressure | .999 | | 0.285 | 3.000 | 634.000 | .836 | .001 |
| Ear noises | .990 | | 2.130 | 3.000 | 634.000 | .095 | .010 |
| Hearing deficits | .986 | | 3.065 | 3.000 | 634.000 | .028 | .014 |
| Visual problems | .962 | | 8.390 | 3.000 | 634.000 | .000 | .038 |
| Sex | .951 | | 10.886 | 3.000 | 634.000 | .000 | .049 |
| Type of vertigo | .970 | | 3.288 | 6.000 | 1268.000 | .003 | .015 |
| Frequency of vertigo | .892 | | 8.228 | 9.000 | 1543.140 | .000 | .037 |
| **Post-hoc univariate ANOVAs for every dependent variable (only significant effects reported)** | | | | | | | |
|  | | **Dependent variable** | | **F** | **p** | **partial η²** | |
| Corrected model | | DHI-Physical-Total | | 8.905 | .000 | .164 | |
|  |  | DHI-Functional-Total | | 12.453 | .000 | .215 | |
|  |  | DHI-Emotional-Total | | 10.290 | .000 | .185 | |
| Headache | | DHI-Physical-Total | | 7.746 | .006 | .012 | |
|  |  | DHI-Functional-Total | | 14.089 | .000 | .022 | |
|  |  | DHI-Emotional-Total | | 11.151 | .001 | .017 | |
| Hearing deficits | | DHI-Physical-Total | | 1.246 | .265 | .002 | |
|  |  | DHI-Functional-Total | | 6.580 | .011 | .010 | |
|  |  | DHI-Emotional-Total | | 8.058 | .005 | .013 | |
| Visual problems | | DHI-Physical-Total | | 8.415 | .004 | .013 | |
|  |  | DHI-Functional-Total | | 20.104 | .000 | .031 | |
|  |  | DHI-Emotional-Total | | 18.187 | .000 | .028 | |
| Sex | | DHI-Physical-Total | | 19.856 | .000 | .030 | |
|  |  | DHI-Functional-Total | | 25.078 | .000 | .038 | |
|  |  | DHI-Emotional-Total | | 15.018 | .000 | .023 | |
| Type of vertigo | | DHI-Physical-Total | | 3.022 | .049 | .009 | |
|  |  | DHI-Functional-Total | | 1.112 | .330 | .003 | |
|  |  | DHI-Emotional-Total | | 6.326 | .002 | .020 | |
| Frequency of vertigo | | DHI-Physical-Total | | 16.906 | .000 | .074 | |
|  |  | DHI-Functional-Total | | 18.288 | .000 | .079 | |
|  |  | DHI-Emotional-Total | | 8.467 | .000 | .038 | |
